# Transcription factors MdEIL1 and MdHY5 integrate ethylene and light signaling to promote chlorophyll degradation in mature apple peels

**DOI:** 10.1093/hr/uhae324

**Published:** 2024-11-21

**Authors:** Li-Xian Li, San-Kui Yang, Yue Fang, Zhi-Meng Wu, Hua-Ying Ma, Shuo Wang, Dan Li, Shou-Qian Feng

**Affiliations:** College of Horticulture Science and Engineering, Shandong Agricultural University, Tai’an, Shandong 271018, China; College of Horticulture Science and Engineering, Shandong Agricultural University, Tai’an, Shandong 271018, China; College of Horticulture Science and Engineering, Shandong Agricultural University, Tai’an, Shandong 271018, China; College of Horticulture Science and Engineering, Shandong Agricultural University, Tai’an, Shandong 271018, China; College of Horticulture Science and Engineering, Shandong Agricultural University, Tai’an, Shandong 271018, China; College of Horticulture Science and Engineering, Shandong Agricultural University, Tai’an, Shandong 271018, China

## Abstract

Although it is well established that ethylene and light stimulate the process of chlorophyll degradation in mature apple peels, there is still a need for further exploration of the molecular mechanisms that regulate this process. This study identified *MdEIL1* and *MdHY5* as promoters of the chlorophyll degradation pathway in apple peels, activated by ethylene and light. Physiological and molecular tests demonstrated that MdEIL1 and MdHY5 are responsible for activating the expression of genes associated with chlorophyll degradation, including *MdERF17*, *MdNYC1*, *MdPPH*, and *MdPAO*. Furthermore, the interaction between MdEIL1 and MdHY5 proteins enhances their regulatory activity on the target gene *MdERF17*. Moreover, MdEIL1 binds to the promoter of *MdHY5*, resulting in the upregulation of its expression, which is further enhanced in the presence of the MdEIL1-MdHY5 protein complex. These findings indicate that MdEIL1-MdHY5 module acts as positive regulator mediating ethylene and light signals that promote chlorophyll degradation in apple peels.

## Introduction

During fruit ripening, various physiological and phenotypic changes occur, including color change, softening, decreased acid content, increased sugar content, and the production of aromatics [1, 2]. One of the critical phenomena during this process is the degreening caused by the progressive degradation of chlorophyll [3, 4]. The degreening significantly impacts the visual quality and commercial value of the fruit, making it an important breeding target for developing new fruit varieties [5, 6]. Therefore, understanding the genetic basis and regulatory pathways involved in chlorophyll degradation is essential for fruit breeding.

Molecular and genetic studies on *Arabidopsis thaliana* and *Oryza sativa* have identified multiple genes associated with chlorophyll (chl) catabolism [7, 8]. The first step in the chl degradation pathway involves the conversion of chl b to chl a, catalyzed by chl b reductase (NYC1/NOL) and 7-hydroxymethyl chl a reductase (HCAR) through two reductive reactions [8–10]. This process leads to the removal of the Mg atom from chl a, forming pheophytin a. The phytol tail of pheophytin a is then cleaved by pheophytin pheophorbide hydrolase (PPH), resulting in pheophorbide a [11, 12]. Pheophorbide a subsequently undergoes oxygenolytic ring opening by pheophorbide a oxygenase (PAO) to produce red chl catabolite (RCC), which is further degraded by RCC reductase (RCCR) [13]. Ultimately, the conversion of pheophorbide a to RCC leads to the loss of green coloration.

Recently, the role of plant hormones, including ethylene, abscisic acid (ABA), and jasmonic acid (JA), in regulating chlorophyll degradation has been widely investigated. In particular, ethylene has been well documented for its regulatory role in promoting chlorophyll degradation [14–20]. In 1995, it was discovered that *1-Aminocyclopropane-1-Carboxylic Acid Oxidase 1* (*ACO1)* antisense tomato plants with decreased levels of the ethylene precursor 1-aminocyclopropane-1-carboxylate (ACC) not only produced less ethylene but also exhibited delayed leaf degreening [21]. Similarly, ethylene production in silenced *1-Aminocyclopropane-1-Carboxylic Acid Synthase* (*ACS)* mutants of *A. thaliana* was only 10% of that of the wild type, significantly delaying leaf degreening [22]. Additionally, ethylene-insensitive mutants *ETHYLENE RECEPTOR 1* (*ETR1)*, *ETHYLENE-INSENSITIVE 2 (EIN2)*, and *ETHYLENE-INSENSITIVE 3* (*EIN3)* also exhibit delayed chlorophyll degradation [23–25]. Further research has indicated that EIN3 and EIN3-LIKE 1 (EIL1) are the primary regulatory nodes of ethylene-induced degreening, with EIN3 positively impacting ethylene-mediated chlorophyll degradation [18, 26–28]. Notably, in apples, the insertion of eight serine (Ser) residues in the coding region of *ETHYLENE RESPONSE FACTOR 17* (*MdERF17*) enhances the transcriptional regulation activity of MdERF17 on the promoters of *MdNYC1* and *MdPPH*, thereby accelerating chlorophyll degradation in apple peels [29]. Furthermore, phosphorylation of MdERF17 and MdMPK14-14G further enhances MdERF17’s regulation of chlorophyll degradation genes [30]. Recently, the ethylene-activated U-box type E3 ubiquitin ligase MdPUB24 has been discovered to ubiquitously degrade *MdBEL7* via 26S proteasome, relieving *MdBEL7*’s inhibition of ethylene-mediated chlorophyll degradation in fruit [31].

The transcriptional regulation of *ACS* and *ACO* is one of the key mechanisms controlling ethylene biosynthesis [32]. Light can regulate ethylene biosynthesis through specific photoreceptor-mediated pathways, and several transcription factors have been identified as potential integrators of light signaling and ethylene biosynthesis regulation [32–34]. Additionally, light plays a crucial role in regulating chlorophyll degradation through ethylene signaling. For example, under dark conditions, the level of endogenous ethylene in the leaves of the *pif4* mutant decreased significantly, while it increased in the leaves of the *PIF4*-OX line [35]. Furthermore, when treated with ethylene, *pif3*, *pif4*, and *pif5* mutants exhibited a green phenotype. PIF4 and PIF5 bind to the promoter of *NYC1* to upregulate its transcription [35, 36], indicating that the negative regulation genes *PIF3*/*4*/*5* of the light signal transduction pathway positively influence ethylene-induced leaf degreening. In addition, another key factor in light regulation is the basic leucine zipper (bZIP) transcription factor LONG HYPOCOTYL 5 (HY5), which promotes photomorphogenesis by controlling the transcription of light-induced genes [37]. Although HY5 primarily functions in light signaling, it remains unclear whether it also affects ethylene signaling pathways that regulate chlorophyll degradation. However, in *Arabidopsis*, the *hy5* mutant exhibited key phenotypes such as inhibition of hypocotyl elongation, greening, anthocyanin accumulation, and lateral root formation, which may be associated with the regulation of hormones such as ethylene, cytokinin, and auxin [38–40]. Currently, it is unclear whether gene upregulation via light signaling significantly affects ethylene-regulated chlorophyll degradation. Sometimes, chlorophyll degradation occurs more rapidly under light conditions than in the dark. For instance, mature apples exposed to ample light tend to undergo green color loss faster than those kept in darkness. Since ethylene plays a vital role in chlorophyll degradation during apple fruit ripening, it is possible that light also participates in ethylene-induced chlorophyll degradation in apples. However, the molecular regulatory mechanisms underlying this phenomenon are unclear and require further analysis.

The present study provides insights into the roles of the key transcription factors MdEIL1 and MdHY5 in apple degreening through the coordination of ethylene and light signaling. We demonstrated that *MdEIL1* and *MdHY5* upregulate the expression of genes associated with chlorophyll degradation, specifically *MdERF17*, *MdNYC1*, *MdPPH*, and *MdPAO*, via the interaction of light and ethylene signaling pathways. Additionally, the interaction between MdEIL1 and MdHY5 proteins enhances the regulatory effect on the target genes *MdHY5* and *MdERF17*. Overall, the results offer novel insights into the synergistic effects of ethylene and light-induced chlorophyll degradation in apple peels.

## Results

### Effects of ethylene and light on chlorophyll degradation in apple peels

**Figure 1:**
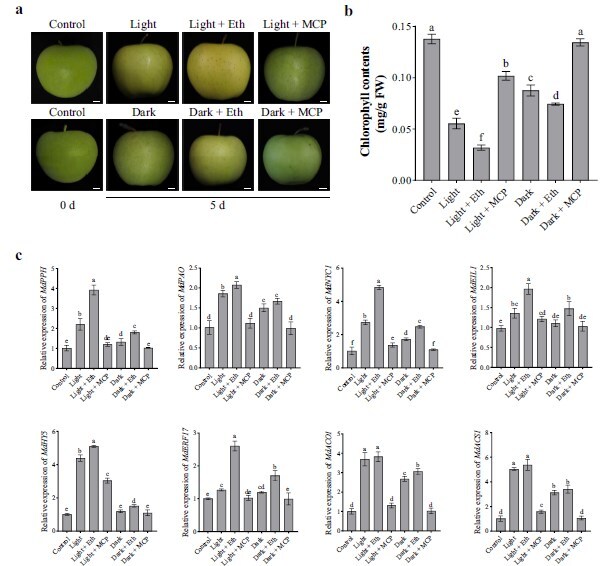
Ethylene and light-induced chlorophyll degradation in apple peels. **a,** Phenotypes of cv. ‘Golden Delicious’ apple fruits exposed to ethylene and 1-MCP under light (20 000 lux) or dark conditions for 5 days. Bars, 1 cm. **b,** Chlorophyll content of cv. ‘Golden Delicious’ apple fruits exposed to ethylene and 1-MCP under light (20 000 lux) or dark conditions for 5 days. **c,** The expression levels of *MdEIL1*, *MdHY5*, *MdERF17*, *MdACO1, MdACS1,* and chlorophyll degradation-related genes of cv. ‘Golden Delicious’ apple fruits exposed to ethylene and 1-MCP under light (20 000 lux) or dark conditions for 5 days. Control, apple fruits at 0 d; Eth and MCP for apples treated with ethephon and 1-MCP, respectively. Data are shown to be the mean ± SD of three biological replicates. Differences among samples were assessed using one-way ANOVA followed by Tukey’s test at *P* < 0.05.

To explore the impacts of ethylene and light signaling on chlorophyll degradation in apple peels, we treated mature cv. ‘Golden Delicious’ apples with ethephon and ethylene inhibitor (1-MCP), and compared their effects on chlorophyll degradation under light and dark conditions. The results indicated that 1-MCP inhibited chlorophyll degradation in apple peels under both light and dark conditions, while ethephon promoted chlorophyll degradation (Fig. 1a and b). Furthermore, chlorophyll degradation in ethephon-treated apple peels under light conditions was significantly faster than under dark conditions (Fig. 1a and b), indicating an additive effect of ethylene and light signaling. To further explore the roles of ethylene and light signaling in chlorophyll degradation, we analyzed the expression levels of key transcription factors (*MdEIL1*, *MdHY5*, and *MdERF17*), chlorophyll degradation-related genes (*MdPPH*, *MdPAO*, and *MdNYC1*), and ethylene biosynthesis-related genes (*MdACO1* and *MdACS1*) [29, 41–44]. The results revealed that the expression patterns of *MdPPH*, *MdPAO*, and *MdNYC1* were consistent with changes in chlorophyll content. In addition, while both *MdEIL1* and *MdHY5* were significantly induced by ethylene and light signaling, *MdEIL1* was primarily induced by ethylene, and *MdHY5* was primarily induced by light. *MdERF17*, however, was coinduced by both light and ethylene, with its expression significantly elevated in the cotreated apple peels. We also found that in apples exposed to light for 5 days, the expression levels of the ethylene biosynthesis genes *MdACO1* and *MdACS1* were significantly upregulated, further indicating that light promotes ethylene synthesis (Fig. 1c). These findings suggest that ethylene and light signaling may jointly regulate chlorophyll degradation in apple peels, with *MdEIL1* and *MdHY5* potentially playing key roles in this process.

### *MdEIL1* promotes the chlorophyll degradation in apple peels

**Figure 2:**
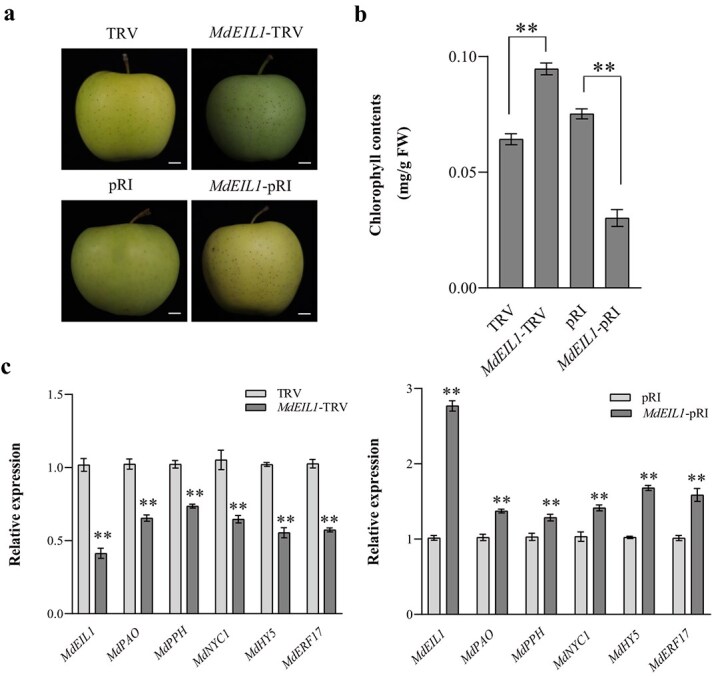
MdEIL1 promotes chlorophyll degradation in cv. ‘Golden Delicious’ apple peels. **a,** Phenotypes of cv. ‘Golden Delicious’ apple fruits with *MdEIL1* silenced or overexpressed. Bars, 1 cm. **b,** Chlorophyll content of cv. ‘Golden Delicious’ apple peels with *MdEIL1* silenced or overexpressed*.*  **c,** The expression levels of *MdHY5* and chlorophyll degradation-related genes in cv. ‘Golden Delicious’ apple peels with *MdEIL1* silenced*.*  **d,** The expression levels of *MdHY5* and chlorophyll degradation-related genes in cv. ‘Golden Delicious’ apple peels with *MdEIL1* overexpressed*.* Data are shown to be the mean ± SD of three biological replicates. Asterisks are thought to be statistically significantly different (^**^  *P* < 0.01), which are conducted on the basis of the Student’s *t*-test.

In further investigating the regulatory role of MdEIL1 in the chlorophyll degradation of apple peels, we conducted a transient transformation experiment using cv. ‘Golden Delicious’ apple fruits. Specifically, we overexpressed and silenced *MdEIL1* in apple peels and measured their chlorophyll content. Our results indicated that, compared to the control group, the chlorophyll content in the apple peels overexpressing *MdEIL1* was significantly reduced, while the chlorophyll content in the apple peels with silenced *MdEIL1* was significantly increased (Fig. 2a and b). To explore whether *MdEIL1* is involved in the ethylene-mediated regulation of chlorophyll degradation in apple peels, we treated both *MdEIL1-*silenced and non-silenced ‘Golden Delicious’ apple fruits with ethephon (1000 mg∙l^−1^). We found that the chlorophyll content in the peels of *MdEIL1*-silenced apples was significantly higher than that of the control when treated with ethephon (Supplemental Fig. S1a and b), further demonstrating that *MdEIL1* plays an essential role in the ethylene regulation of chlorophyll degradation in apple peels.

Compared with the control, *MdHY5*, the key chlorophyll degradation regulator *MdERF17*, and *MdNYC1*, *MdPPH*, and *MdPAO* were significantly upregulated in the apple peels overexpressing *MdEIL1*, while the opposite results were observed in the apple peels with silenced *MdEIL1* (Fig. 2c and d). Moreover, in ethephon-treated cv ‘Golden Delicious’ apple peels, the expression levels of these genes in the *MdEIL1*-silenced apple peels were significantly lower than those in the control group (Supplemental Fig. S1c). Furthermore, we obtained stable apple calli overexpressing *MdEIL1* and found that the expression levels of *MdHY5*, *MdERF17*, *MdNYC1*, *MdPPH*, and *MdPAO* were significantly increased compared to wild-type apple calli (Supplemental Fig. S2). Collectively, these findings suggest that *MdEIL1* may directly or indirectly regulate the transcription of these chlorophyll-related genes and play a positive role in the regulation of ethylene-mediated chlorophyll degradation in apple peels.

### MdEIL1 binds to the *MdERF17* promoter to enhance its expression

**Figure 3:**
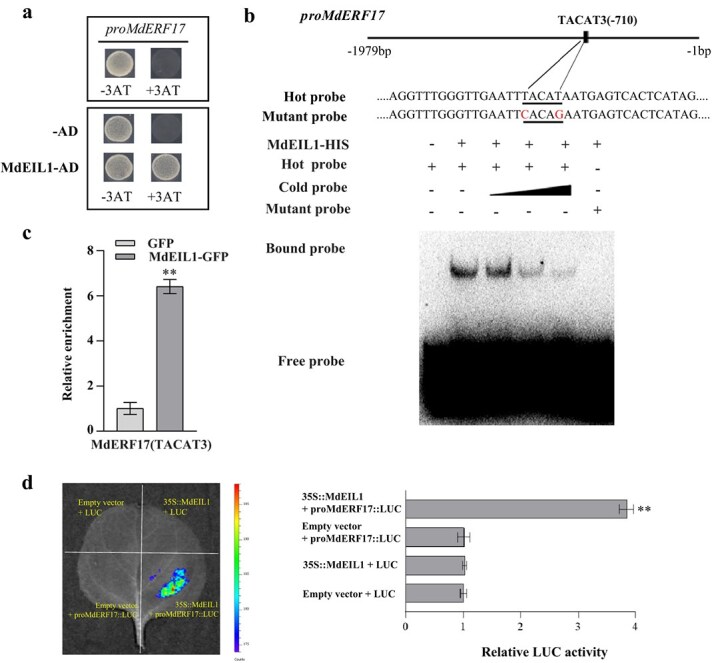
MdEIL1 promotes *MdERF17* expression. **a,** Y1H assays showing the correlation between MdEIL1 and the *MdERF17* promoter. The 3-AT concentration was 120 mM. **b** EMSA analysis indicated that MdEIL1 binds to the TACAT3 motif in the *MdERF17* promoter. The hot probe was a biotin-labeled promoter fragment that contained the TACAT3 motif, and the cold probe was an unlabeled competitive probe (added according to a concentration gradient 25×, 50×, and 100×). Mutation probes were unlabeled hot probes that contained two nucleotide mutations. **c,** ChIP-qPCR indicated that MdEIL1 binds to the *MdERF17* promoter *in vivo*. The ‘Orin’ apple calli overexpressing a GFP tag was applied to be the negative control (NC). Three biological replicates were used. **d** LUC activity analysis showed that MdEIL1 activates the *MdERF17* promoter. Data are shown to be mean ± SD of three biological replicates. Asterisks are thought to be statistically significantly different (^**^  *P* < 0.01), which were conducted on the basis of the Student’s *t*-test.

Based on above research findings (Fig. 2), we hypothesized that MdEIL1 may directly regulate *MdERF17*. Promoter element analysis identified three potential MdEIL1 binding motifs (TACAT1–3) in the *MdERF17* promoter, suggesting that MdEIL1 may bind to the *MdERF17* promoter. To test this hypothesis, yeast one-hybrid (Y1H) analysis was performed. Specifically, we cotransformed Y187 yeast cells with the *MdEIL1*-pGADT7 and pro*MdERF17*-pHIS2 plasmids and grew them in SD (−Trp/−Leu/−His) medium containing 120 mM 3-AT. According to our results, the Y187 yeast cells cotransformed with these plasmids could grow normally under these conditions (Fig. 3a). Furthermore, electrophoretic mobility shift assay (EMSA) experiments show that MdEIL1 can bind the TACAT3 motif in the *MdERF17* promoter (Fig. 3b; Supplemental Fig. S3). To confirm these results *in vivo*, ChIP-qPCR (Chromatin Immunoprecipitation followed by quantitative Polymerase Chain Reaction) analysis was conducted, which showed that promoter fragments containing the TACAT3 motif were more enriched in *MdEIL1*-overexpressing calli compared to the control (Fig. 3c). Then, this study focused on investigating the impact of MdEIL1 on the activity of the *MdERF17* promoter using a luciferase (LUC) assay and found that MdEIL1 significantly activated the *MdERF17* promoter (Fig. 3d). These findings suggest that *MdERF17* functions as a downstream gene of MdEIL1.

### *MdHY5* promotes the chlorophyll degradation in apple peels

**Figure 4:**
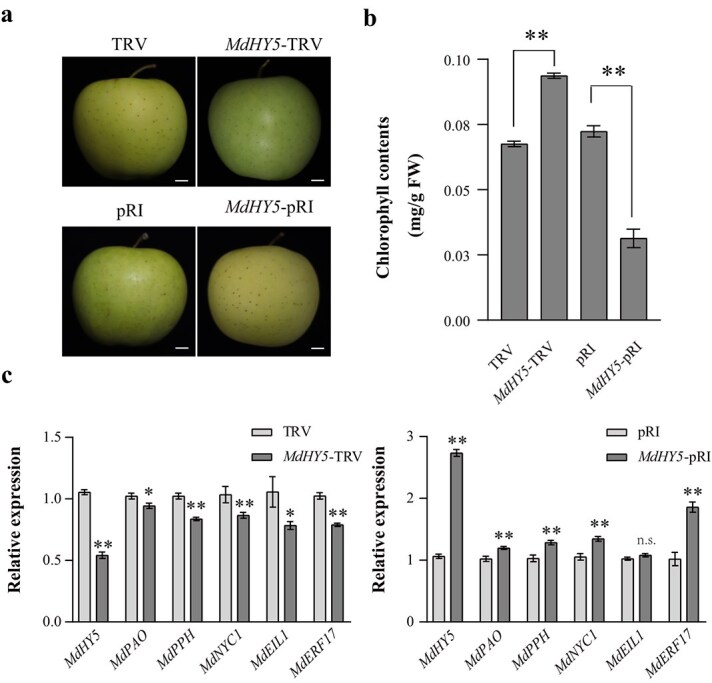
*MdHY5* promotes chlorophyll degradation in cv. ‘Golden Delicious’ apple peels. **a,** Phenotypes of cv. ‘Golden Delicious’ apple fruits with *MdHY5* silenced or overexpressed*.* Bars, 1 cm. **b,** Chlorophyll content of cv. ‘Golden Delicious’ apple peels with *MdHY5* silenced or overexpressed*.*  **c,** The expression level of *MdERF17* and chlorophyll degradation-related genes in cv. ‘Golden Delicious’ apple peels with *MdHY5* silenced*.*  **d,** The expression level of *MdERF17* and chlorophyll degradation-related genes in cv. ‘Golden Delicious’ apple peels with *MdHY5* overexpressed*.* Data are represented to be the mean ± SD of three biological replicates. Asterisks suggest statistically significant differences (^*^  *P* < 0.05, ^**^  *P* < 0.01), which were conducted using the Student’s *t*-test. n.s., no significant difference.

Expression patterns showed that *MdHY5* might participate in apple chlorophyll degradation (Figs. 1 and 2). To investigate the impact of *MdHY5* on chlorophyll degradation in apple peels, we conducted transient overexpressing and silencing *MdHY5* in cv. ‘Golden Delicious’ apples. Compared to control, the overexpression of *MdHY5* significantly reduced chlorophyll content in apple peels, whereas silencing *MdHY5* significantly increased the chlorophyll content (Fig. 4a and b). Real-time quantitative polymerase chain reaction (RT-qPCR) analysis demonstrated that overexpression of *MdHY5* notably upregulated the expression levels of *MdERF17*, *MdNYC1*, *MdPPH*, and *MdPAO* in apple peels, while silencing *MdHY5* significantly downregulated these genes (Fig. 4c and d). Subsequently, we generated apple calli overexpressing *MdHY5* and observed that the expression levels of *MdERF17*, *MdNYC1*, *MdPPH*, and *MdPAO* were significantly increased compared to wild type (Supplemental Fig. S4), further highlighting that *MdHY5* transcriptionally promotes chlorophyll degradation in apple peels.

Our previous results showed that light can significantly promote chlorophyll degradation induced by ethephon. To further confirm whether *MdHY5* participates in this process, we transiently silenced *MdHY5* in cv. ‘Golden Delicious’ apple fruits and exposed them to ethephon (1000 mg∙l^−1^). The results showed that, after ethephon treatment, the chlorophyll content in the apple peels with silenced *MdHY5* expression was significantly higher than that in the control (Supplemental Fig. S5a and b). Additionally, the expression levels of *MdERF17*, *MdNYC1*, *MdPPH*, and *MdPAO* in the ethephon-treated apple peels with silenced *MdHY5* expression were significantly lower compared to the control (Supplemental Fig. S5c). These findings suggest that *MdHY5* exerts an important role in light- and ethylene-induced chlorophyll degradation in apple peels.

### MdHY5 activates the expression of *MdERF17*

**Figure 5:**
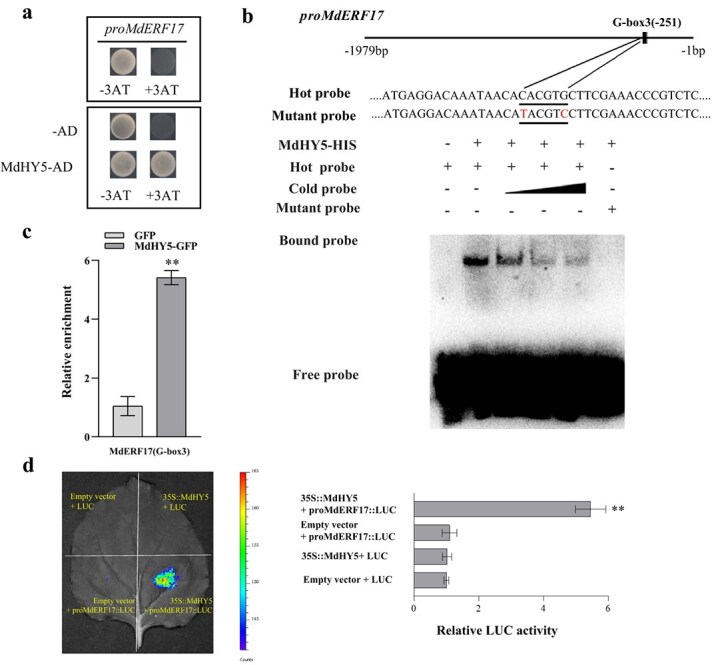
MdHY5 activates *MdERF17* expression. **a,** Y1H assays showed the correlation between MdHY5 and the *MdERF17* promoter. The 3-AT concentration was 120 mM. **b,** EMSA analysis indicated that MdHY5 binds to the G-box3 motif in the *MdERF17* promoter. The hot probe was a biotin-labeled promoter fragment that contained the G-box3 motif, and the cold probe was an unlabeled competitive probe (added according to a concentration gradient 25×, 50×, and 100×). Mutation probes were unlabeled hot probes including two nucleotide mutations. **c,** ChIP-qPCR assays suggested that MdHY5 binds to the *MdERF17* promoter *in vivo*. The ‘Orin’ apple calli overexpressing a GFP tag was applied to be the NC. Three biological replicates were used. **d,** LUC activity analysis showed that MdHY5 activates the *MdERF17* promoter. Data are shown to be mean ± SD of three biological replicates. Asterisks are thought to be statistically significantly different (^**^  *P* < 0.01), which were conducted with the use of the Student’s *t*-test.

We observed the expression levels of *MdERF17* in apple peels with overexpressed and silenced *MdHY5*, as well as in calli overexpressing *MdHY5* (Fig. 4c and d; Supplemental Fig. S4), leading us to speculate that MdHY5 may regulate *MdERF17* expression. In addition, we identified three potential binding motifs (G-box1–3) for MdHY5 within the *MdERF17* promoter, suggesting that MdHY5 might bind to the *MdERF17* promoter. With the aim of testing this hypothesis, we conducted Y1H assays and discovered that Y187 yeast cells cotransformed with *MdHY5*-pGADT7 and pro*MdERF17*-pHIS2 plasmids could grow normally in SD (−Trp/−Leu/−His) medium containing 120 mM 3-AT (Fig. 5a). Furthermore, EMSA indicated that MdHY5 specifically binds to the G-box3 motif in the *MdERF17* promoter and not to the other two G-box motifs (Fig. 5b; Supplemental Fig. S6). Further ChIP-qPCR analysis revealed that the DNA enrichment level of the *MdERF17* promoter containing G-box3 in calli overexpressing *MdHY5* was significantly higher compared to the control group (Fig. 5c). LUC assays also showed that the fluorescence intensity of tobacco leaves cotransfected with 35S::*MdHY5* and pro*MdERF17*::LUC was significantly higher than that of tobacco leaves transfected with pro*MdERF17*::LUC alone (Fig. 5d), implying that MdHY5 positively regulates the activity of the *MdERF17* promoter. These findings demonstrate that MdHY5 can bind to the *MdERF17* promoter and activate its expression.

### MdEIL1 positively regulates *MdHY5* expression

**Figure 6:**
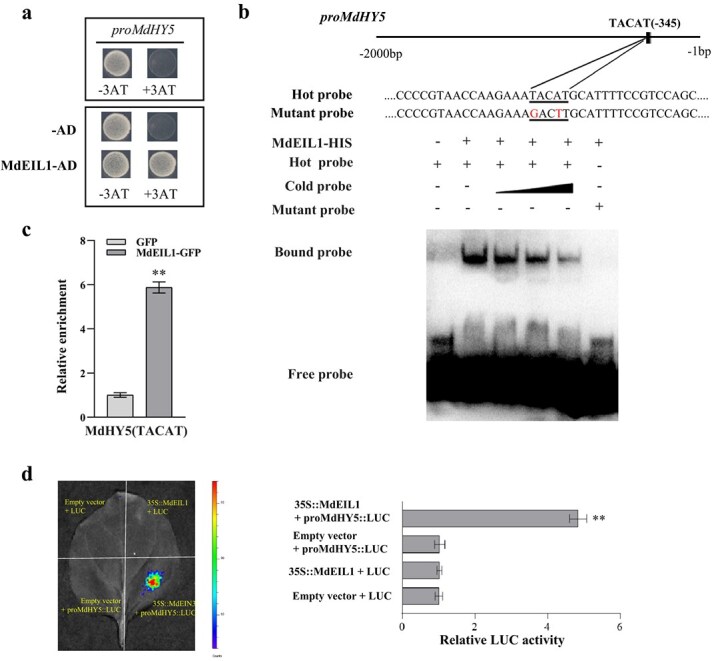
MdEIL1 activates *MdHY5* expression. **a,** Y1H assays showed the correlation between MdEIL1 and the *MdHY5* promoter. The 3-AT concentration was 100 mM. **b,** EMSA analysis showed that MdEIL1 binds to the TACAT motif in the *MdHY5* promoter. The hot probe was a biotin-labeled promoter fragment that contained the TACAT motif, and the cold probe was an unlabeled competitive probe (added according to a concentration gradient 25×, 50×, and 100×). Mutation probes were unlabeled hot probes that contained two nucleotide mutations. **c,** ChIP-qPCR assays revealed that MdEIL1 binds to the *MdHY5* promoter *in vivo*. The ‘Orin’ apple calli overexpressing a GFP tag was applied to be the NC. Three biological replicates were used. **d,** LUC activity analysis indicated that MdEIL1 activates the *MdHY5* promoter. Data are shown to be the mean ± SD of three biological replicates. Asterisks are thought to be statistically significantly different (^**^  *P* < 0.01), which were conducted on the basis of the Student’s *t*-test.

Obviously, the expression level of *MdHY5* was significantly increased in apple peels and calli overexpressing *MdEIL1* (Fig. 2c and d; Supplemental Fig. S2), suggesting a direct regulatory relationship between the two genes. Y1H assay showed that Y187 yeast cells cotransformed with MdEIL1-pGADT7 and proMdHY5-pHIS2 constructs grew normally on SD (−Trp/−Leu/−His) medium containing 100 mM 3-AT (Fig. 6a), indicating that MdEIL1 binds to the *MdHY5* promoter. Further analysis of the *MdHY5* promoter revealed a potential MdEIL1 binding element, TACAT. EMSA and ChIP-qPCR results demonstrated that MdEIL1 specifically binds to the TACAT motif in the *MdHY5* promoter (Fig. 6b and c).

Then, LUC assays were adopted for exploring the regulatory relationship between MdEIL1 and *MdHY5*. The results showed that the fluorescence intensity of tobacco leaves cotransfected with 35S::*MdEIL1* and pro*MdHY5*::LUC was significantly higher than that of tobacco leaves transfected with pro*MdHY5*::LUC alone (Fig. 6d), proving that MdEIL1 positively regulates the activity of the *MdHY5* promoter. Collectively, multiple lines of evidence strongly indicate that MdEIL1 activates the *MdHY5* promoter and increases *MdHY5* expression*.*

### MdEIL1 interacts with MdHY5

**Figure 7:**
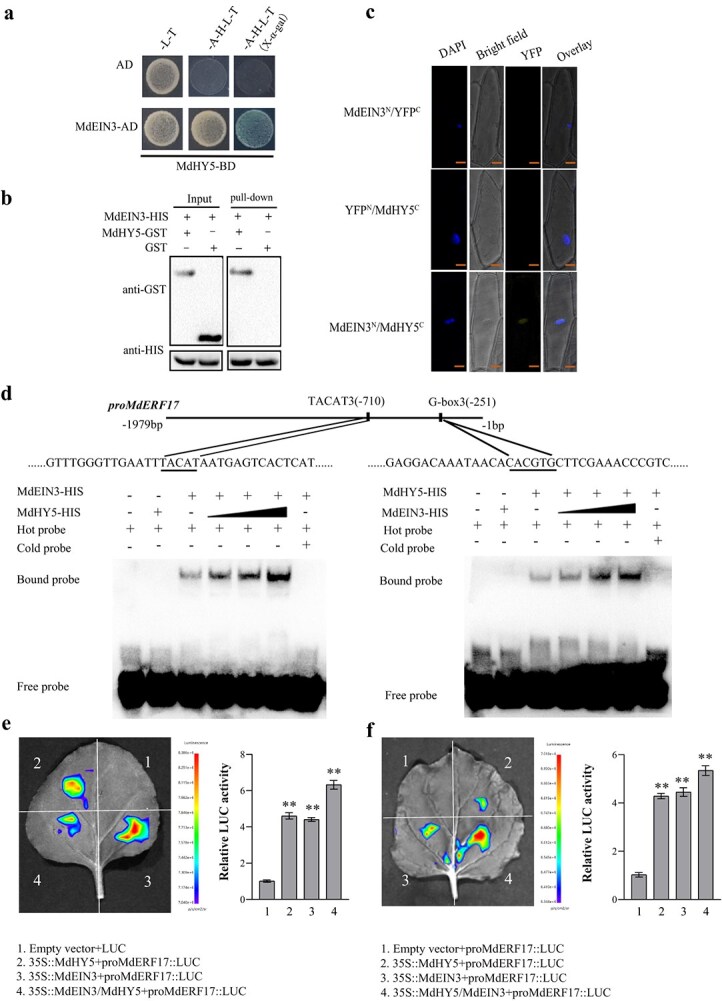
Correlation between MdEIL1 and MdHY5. **a,** Y2H assays indicated the correlation between MdEIL1 and MdHY5. The empty pGADT7 vector (AD) acted as an NC. **b,** Pull-down experiments indicated that MdEIL1 interacts with MdHY5. The GST antibody depicted protein bands, suggesting that MdEIL1-HIS pulled down MdHY5-GST. **c,** BiFC assay represented the correlation between MdEIL1 and MdHY5 *in vivo*. Bars, 50 μm. **d,** EMSA analysis showed that adding MdHY5 (added according to a concentration gradient 25×, 50×, and 100×) enhanced transcriptional activation of the *MdERF17* promoter by MdEIL1 and adding MdEIL1 (added according to a concentration gradient 25×, 50×, and 100×) enhanced transcriptional activation of the *MdERF17* promoter by MdHY5. **e–f** LUC activity analysis showed that the correlation between MdEIL1 and MdHY5 increases the transcriptional activation of MdEIL1 on *MdERF17*(**e**) and MdHY5 on *MdERF17*(**f**) promoter. Data are shown to be the mean ± SD of three biological replicates. Asterisks suggest statistically significant differences (^**^  *P* < 0.01), which were conducted with the use of the Student’s *t*-test.

Protein interactions play a crucial role in integrating hormone signals. To investigate the potential protein interaction between MdEIL1 and MdHY5, we conducted yeast two-hybrid (Y2H) assays. The results indicated that yeast cells cotransformed with MdEIL1-pGADT7 and MdHY5-pGBDT7 could grow normally on SD/−Trp/−Leu/−His/−Ade medium, while the control group could not grow, indicating that MdEIL1 interacted with MdHY5 in yeast (Fig. 7a). Subsequently, pull-down assays revealed that MdEIL1-HIS could pull down MdHY5-GST, further demonstrating the interaction between MdEIL1 and MdHY5 (Fig. 7b). In addition, BiFC assays revealed a stable yellow fluorescent protein (YFP) signal in the nucleus of onion cells coexpressing MdEIL1-pSPYNE and MdHY5-pSPYCE (Fig. 7c), demonstrating that MdEIL1 can interact with MdHY5 *in vivo*.

To determine whether the interaction between MdEIL1 and MdHY5 proteins influences their upregulation of the *MdHY5* and *MdERF17* promoters, we conducted EMSA and LUC assays. The results showed that the coexistence of MdEIL1 and MdHY5 significantly enhanced their binding and activation of the *MdHY5* and *MdERF17* promoters, compared to MdEIL1 or MdHY5 alone, respectively (Fig. 7d–f; Supplemental Fig. S7). To further investigate the synergistic effect of MdEIL1 and MdHY5 on chlorophyll degradation in apples, we conducted a transient infection assay coexpressing *MdEIL1* and *MdHY5* in apple fruits. The results showed that co-overexpression of *MdEIL1* and *MdHY5* in apples led to significantly greater chlorophyll loss in the fruit peels compared to fruits overexpressing either *MdEIL1* or *MdHY5* alone (Supplemental Fig. S8a and b). Measurement of chlorophyll content revealed a corresponding significant reduction in the chlorophyll levels in the peel of apples co-overexpressing *MdEIL1* and *MdHY5*. These results indicate that MdEIL1 and MdHY5 coregulate chlorophyll degradation in apple fruits (Supplemental Fig. S8b).

## Discussion

Chlorophyll degradation is a vital characteristic of fruit ripening and leaf senescence, tightly regulated by environmental factors and hormones [45, 46]. Among these, ethylene is the primary hormone involved in regulating chlorophyll degradation [47–49]. While light is generally believed to promote chlorophyll synthesis in photosynthetic tissues [50], it can also promote chlorophyll degradation through photobleaching [51, 52]. However, whether light is involved in chlorophyll degradation through a molecular pathway in certain plant tissues remains unknown. Our results suggest that light enhances the function of ethylene in regulating chlorophyll degradation in apple fruit. Through a series of physiological and molecular experiments, we found that ethylene and light upregulate the expression of *MdERF17* via *MdEIL1* and *MdHY5*, thereby promoting chlorophyll degradation in apple peels.

The ethylene-responsive transcription factor *MdERF17* can regulate chlorophyll degradation and degreening of apple peels (Supplemental Fig. S10) [29]. Recent studies have shown that MdMPK4-14G positively regulates apple peel degreening by phosphorylating MdERF17 under dark conditions [30]. Notably, the rate of chlorophyll degradation in mature apple peels was faster in the presence of light compared to dark conditions. The expression level of *MdERF17* was upregulated by both light and ethephon treatment (Fig. 1c). Furthermore, MdEIL1 and MdHY5 have been shown to bind to the *MdERF17* promoter and activate its expression (Fig. 3, Fig. 5), indicating that both ethylene and light promote chlorophyll degradation in apple peels at the transcriptional level through *MdERF17*. As a result, *MdERF17* is a key node in this regulatory pathway.

*EIN3* is an important positive regulator of chlorophyll degradation in response to ethylene, influencing the process through multiple pathways. First, EIN3 directly binds to the promoters of *NYC1*, *NYE1*, and *PAO* to activate their expression [18]. Second, EIN3 positively regulates *ORE1* expression either directly or indirectly through the negative regulation of *miR164*, thereby indirectly affecting the expressions of *NYE1*, *NYC1*, *NOL*, and *PAO* [26–28]. In this study, we identified two pathways in apples that promote chlorophyll degradation via *EIL1*, a homolog of *EIN3*. One pathway is that MdEIL1 indirectly upregulates the expressions of *MdNYC1*, *MdPPH*, and *MdPAO* by positively regulating *MdERF17*. In the second pathway, MdEIL1 enhances the expressions of *MdERF17*, *MdNYC1*, *MdPPH*, and *MdPAO* by positively regulating *MdHY5*. Interestingly, we found that MdEIL1 interacts with MdHY5 to enhance its regulatory activity on *MdERF17*, suggesting a convergence of these two pathways.

It is indicated that *MdHY5* positively regulates chlorophyll degradation in apple skin. In *Arabidopsis*, the negative regulators of the light signal transduction pathway, *AtPIF4* and *AtPIF5*, upregulate *AtNYC1* expression, which is also a positive regulator of chlorophyll degradation [35, 36]. The use of different experimental materials and their complex chlorophyll degradation regulatory networks might illustrate why the positive regulator of light signaling, *MdHY5*, and the negative regulators of light signaling, *AtPIF4* and *AtPIF5*, all function as positive regulators for chlorophyll degradation. In addition, there is evidence that *AtPIF4* and *AtPIF5* are involved in ethylene-induced chlorophyll degradation [14, 35]. In this study, we discovered that MdEIL1 binds to *MdHY5* promoter and activates *MdHY5*. Chlorophyll content in *MdHY5*-TRV apple skin treated with ethephon increased relative to control (Supplemental Fig. S5b), suggesting that *MdHY5* exerts a critical effect on promoting ethylene-induced chlorophyll degradation. Furthermore, we found that MdHY5 does not affect *MdEIL1* expression. However, there is currently no evidence suggesting that *MdHY5* regulates ethylene signal transduction.

Ethylene and light play vital roles in anthocyanin synthesis in apples [42, 43, 53]. *MdEIL1*, *MdERF17*, and *MdHY5* are known as positive regulatory genes for anthocyanin synthesis [29, 42, 54]. Interestingly, this study also shows that *MdEIL1*, *MdERF17*, and *MdHY5* positively regulate light- and ethylene-induced chlorophyll degradation in apple peels. Since both anthocyanin synthesis and chlorophyll degradation occur during fruit ripening, future studies are needed to explore the correlation between light and ethylene in promoting these processes in apple peels. This will provide a better understanding of the complex regulatory network behind the formation of fruit appearance quality during ripening.

In this study, we found that ethylene promotes the degradation of chlorophyll in mature apple fruit peels. Additionally, light enhances ethylene-induced chlorophyll degradation in mature apple peels, leading to rapid fruit degreening. Further research revealed that the light signal transduction factor *MdHY5* interacts with the ethylene signal transduction factors *MdEIL1* and *MdERF17*, thereby facilitating the expression of chlorophyll degradation-related genes and chlorophyll degradation. Unlike mature apples, young fruits have lower ethylene production but possess a strong capacity for chlorophyll synthesis in their peels. While ethylene treatment at this stage does promote chlorophyll degradation in the peel, the enhancing effect of light on ethylene-induced chlorophyll degradation in young apple peels is not of significance (Fig. 1, Supplemental Fig. S10). Given the significant physiological differences between mature and young apples, cross-talk from other hormones and signaling pathways probably influences how light and ethylene are perceived during the chlorophyll degradation process in apple peels.

**Figure 8:**
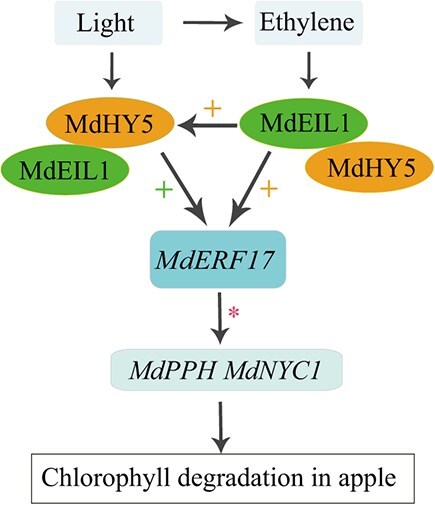
Ethylene and light promote chlorophyll degradation in apple peels through *MdEIL1* and *MdHY5*. Light promoted ethylene biosynthesis, and ethylene and light activated the expression of *MdEIL1* and *MdHY5*. These two transcription factors, MdEIL1 and MdHY5, cobind to the *MdERF17* promoter and directly upregulate the expression of *MdERF17*. MdEIL1 binds to the *MdHY5* promoter to activate the expression of *MdHY5* and indirectly regulates the expression of *MdERF17* through this pathway. At the same time, the MdEIL1-MdHY5 complex formed by these two proteins promoted the expression of *MdHY5* and *MdERF17*. The upregulated MdERF17 further promoted the expression of *MdPPH* and *MdNYC1*, and ultimately accelerated the degradation of chlorophyll in apple peels. The plus sign shows the enhanced impacts of the MdEIL1*-*MdHY5 protein complex on target genes. The asterisk indicates that the expression of *MdPPH* and *MdNYC1* has been reported to be directly regulated by MdERF17 [29].

Based on our findings, we propose a model describing the important role of *MdEIL1* and *MdHY5* in ethylene- and light-induced chlorophyll degradation in apple peels. Light promotes ethylene biosynthesis, and ethylene and light activate *MdEIL1* and *MdHY5*, which cobind to the *MdERF17* promoter, upregulating *MdERF17* expression and thereby promoting chlorophyll degradation. In addition, MdEIL1 binds to the *MdHY5* promoter to upregulate its expression in a positive feedback system, indirectly regulating the expression of *MdERF17*. Collectively, the interaction between MdEIL1 and MdHY5 enhances the transcriptional regulatory function of the MdEIL1-MdHY5 complex, thereby promoting *MdERF17* expression and chlorophyll degradation in apple peels (Fig. 8).

## Material and methods

### Plant materials and treatments

Experiments were conducted using cv. ‘Golden Delicious’ apple fruits harvested at 56 (young fruits) and 135 (mature fruits) days postflowering. The fruits from both periods were classified into three groups, with eight fruits per group. The first group served as a control and received no treatment. The second group was soaked in ethephon (1000 mg∙l^−1^) for 1 min, and the third group was treated with 1-methylcyclopropene (1-MCP, 1 μl∙l^−1^). All three groups were exposed to either 20 000 lux light or darkness at 24°C. After 5 days, peels were harvested for gene expression analysis and chlorophyll content determination. Three biological replicates were used for each group.

The cv. ‘Orin’ apple calli were cultured on MS solid medium added with 1.5 mg∙l^−1^ 2,4-D and 0.4 mg∙l^−1^ 6-BA.

### Determination of chlorophyll content in apple peels

The chlorophyll content was determined following Jung’s [55] protocol with slight modifications. Briefly, 1 g of liquid nitrogen-ground fruit peel powder was placed in a precooled test tube containing 4 ml of 80% acetone and extracted in the dark at 4°C for 24 h with frequent shaking. The extract was then centrifuged at 5000 rpm for 10 min, and the absorbance values at OD663 nm and OD645 nm were measured from the supernatant. Three biological replicates were measured for each sample.

### RNA extraction and RT-qPCR analysis

Following the manufacturer’s instructions, total RNA was extracted using a plant RNA extraction kit (Tiangen, Beijing, China). First-strand cDNA synthesis was performed by adopting the RevertAid™ first-strand cDNA synthesis kit (TransGen, Beijing) [56]. RT-qPCR was carried out on an iCycler iQ5 system (Bio-Rad Laboratories, CA, USA). The internal reference gene used was *MdActin*, and the relative expression levels of the target genes were calculated using the 2^-ΔΔCt^ method [57]. Each sample was analyzed in triplicate. The primers adopted for RT-qPCR were synthesized by Sangon Biotech (Shanghai, China) and are presented in Table S1.

### Generation of transgenic apple calli

To generate the overexpression vectors *MdEIL1*-pRI and *MdHY5*-pRI, the codon-free *MdEIL1* and *MdHY5* CDSs were inserted into the pRI101 vector. The resulting recombinant plasmids were then transformed into *Agrobacterium tumefaciens* strain LBA4404 for genetic transformation of the cv. ‘Orin’ apple calli [58]. Each successfully transformed apple calli line was applied as a biological replicate.

### Fruit instantaneous transformation

The *MdEIL1*-pRI, *MdHY5*-pRI, and *MdERF17*-pRI expression vectors were generated using the above-described procedure. The CDSs for *MdEIL1* (459 bp), *MdHY5* (360 bp), and *MdERF17* (381 bp) were cloned in pTRV2 vector to produce *MdEIL1*-pTRV2, *MdHY5*-pTRV2, and *MdERF17*-pTRV2. The *Agrobacterium* GV3101 was transformed with the recombinant plasmids, and the transformed *A. tumefaciens* solution was vacuum infiltrated into ‘Golden Delicious’ apple fruits at −70 kPa [59]. The treated fruits were placed in darkness overnight and later kept for 5 days at 24°C, 20 000 lux. Three biological replicates were utilized, with each replicate consisting of eight apple fruits.

### Yeast one-hybrid

The promoter sequences of *MdHY5* (−2000 bp) and *MdERF17* (−1979 bp) were cloned into the pHIS2 vector. The *MdEIL1* and *MdHY5* CDSs were cloned into the pGADT7 vector. Then, the recombinant plasmids were transformed into yeast strain Y187 and screened on SD/−Trp/−Leu/−His medium including 3-AT. The empty pGADT7 plasmid was used as the control.

### Electrophoretic mobility shift assay

The *MdEIL1* and *MdHY5* CDSs were cloned into the pET32a vector. In order to produce His fusion proteins, the recombinant plasmids were introduced into *Escherichia coli* BL21 (DE3) cells [60]. EMSA experiments were conducted following previously established methods using the Light Shift Chemiluminescent EMSA kit (Thermo Scientific, Waltham, MA, USA) [61, 62]. The probe was synthesized by Biotechnology (Shanghai, China).

### ChIP-qPCR analysis

*MdEIL1-* and *MdHY5*-overexpressing ‘Orin’ apple calli with GFP tags were acquired. The enriched DNA fragments were detected based on the EZ-ChIP chromatin immunoprecipitation kit (Millipore/Upstate, MA, USA), with the calli of the ‘Orin’ apple overexpressing GFP sequence as the control [63].

### Yeast two-hybrid

The *MdHY5* CDS was inserted into the pGBKT7 vector and the *MdEIL1* CDS was inserted into the pGADT7 vector. The *MdHY5*-pGBKT7 and *MdEIL1*-pGADT7 recombinant plasmids were cotransformed into yeast strain Y2H and later screened on (SD/−Trp/−Leu/−His/−Ade) medium. Meanwhile, yeast cells were inoculated on medium containing X-α-Gal (SD/−Trp/−Leu/−His/−Ade) for color development.

### BiFC assay

The codon-free *MdEIL1* and *MdHY5* CDSs were inserted into pSPYNE and pSPYCE vectors. The *MdEIL1*-pSPYNE and *MdHY5*-pSPYCE recombinant plasmids were transformed into *A. tumefaciens* strain GV3101 [64]. YFP signal was detected under a DS-Ri2 confocal laser scanning microscope (Olympus BX53F, Tokyo, Japan).

### Pull-down assay

The *MdEIL1* CDS was cloned into the pET-32a (+) vector to generate His-tagged fusion protein. The *MdHY5* CDS was cloned into the pGEX-4 T-1 vector with the purpose of generating GST-tagged fusion protein. The mixture of purified MdEIL1-HIS protein and MdHY5-GST protein was subject to incubation with His-tagged bait protein at 4°C for 12 h. Anti-His and anti-GST antibodies (Clontech, Palo Alto, CA, USA) were adopted for performing western blot analysis.

### LUC analysis

The *MdEIL1* and *MdHY5* CDSs were inserted into pGreenII62-SK as effectors. The *MdHY5* and *MdERF17* promoter sequences were recombined into pGreenII0800-LUC plasmid as reporter genes. The recombinant plasmid was transformed into tobacco leaves. An *in vivo* imaging analysis instrument (NightOWL II LB983, Berthold, Germany) was employed to perform luminescence detection [65].

## Supplementary Material

Web_Material_uhae324

## Data Availability

Results supporting our results can be obtained in supplementary materials of this study.
