# Supplementary material for: Transcription factors MdEIL1 and MdHY5 integrate ethylene and light signaling to promote chlorophyll degradation in mature apple peels
Source: Hortic Res. 2024 Nov 21;12(3):uhae324. doi: 10.1093/hr/uhae324 (PMC11997652; doi:10.1093/hr/uhae324)
Supplement: Web_Material_uhae324 [file web_material_uhae324.zip › FigS7.pdf]

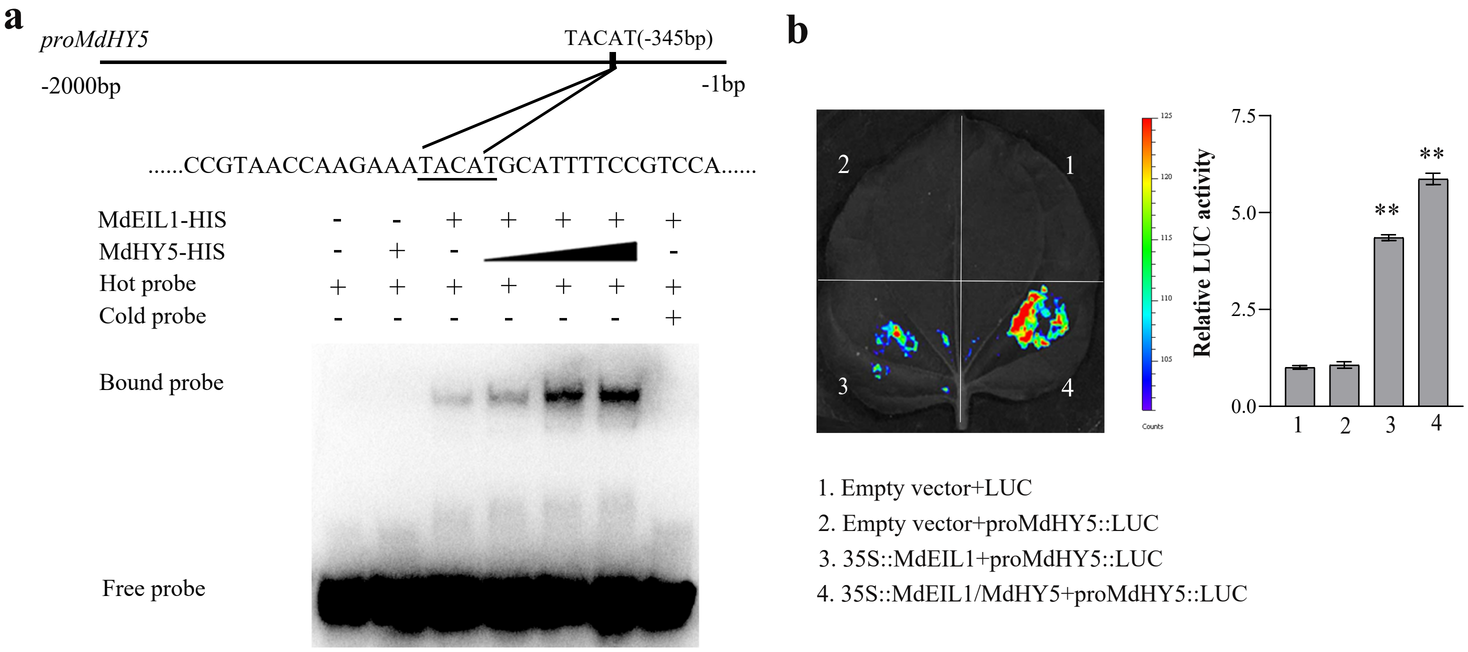
a
proMdHY5
TACAT(-345bp)
-2000bp
-1bp
......CCGTAACCAAGAAATACATGCATTTTCCGTCCA......
MdEIL1-HIS
MdHY5-HIS
Hot probe
Cold probe
Bound probe
Free probe
b
Counts
Relative LUC activity
7.5
5.0
2.5
0.0
1
2
3
4
**
**
1. Empty vector+LUC
2. Empty vector+proMdHY5::LUC
3. 35S::MdEIL1+proMdHY5::LUC
4. 35S::MdEIL1/MdHY5+proMdHY5::LUC
