# Supplementary figures and images for: Transcription factors MdEIL1 and MdHY5 integrate ethylene and light signaling to promote chlorophyll degradation in mature apple peels
Source: Hortic Res. 2024 Nov 21;12(3):uhae324. doi: 10.1093/hr/uhae324 (PMC11997652; doi:10.1093/hr/uhae324)

**a**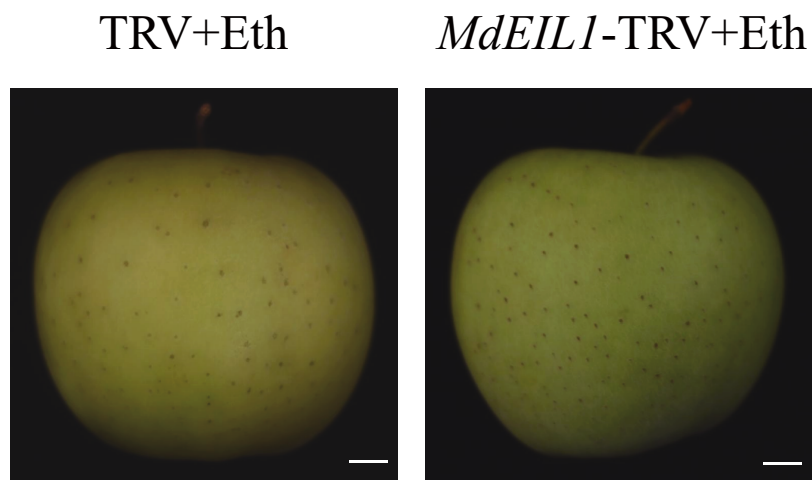**b**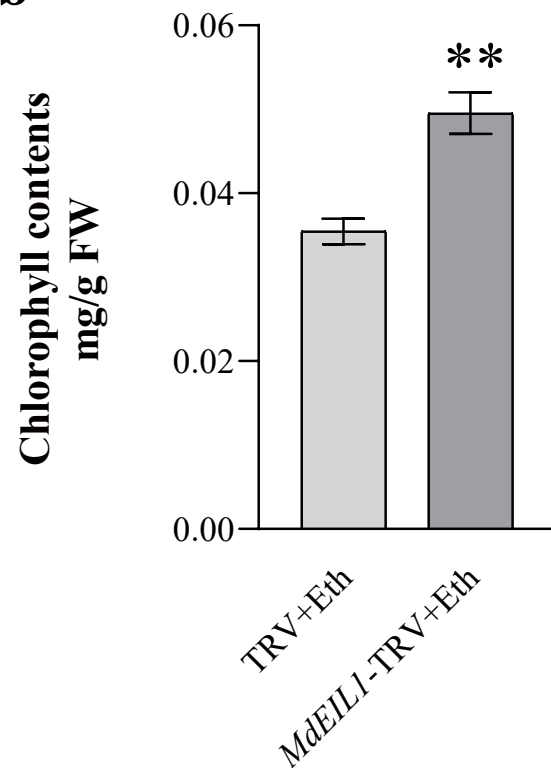**c**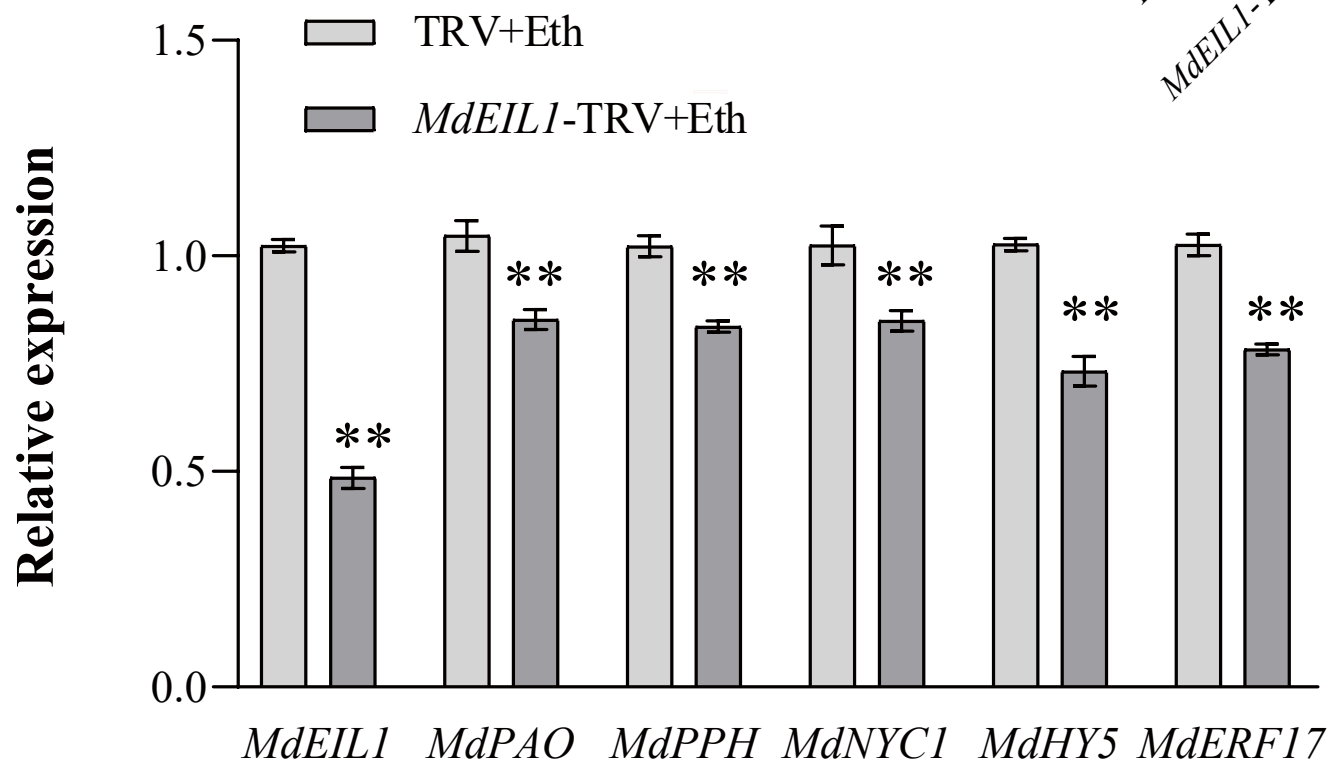

Supplement: Web_Material_uhae324 [file web_material_uhae324.zip › FigS1.pdf]

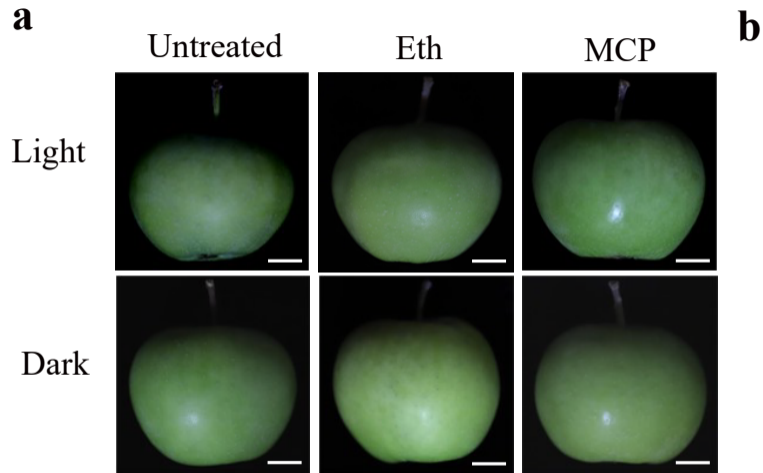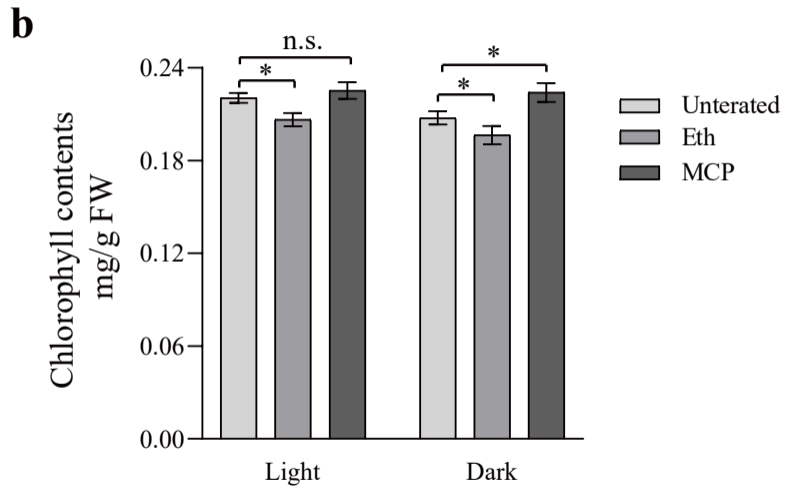

Supplement: Web_Material_uhae324 [file web_material_uhae324.zip › FigS10.pdf]

**Relative expression**

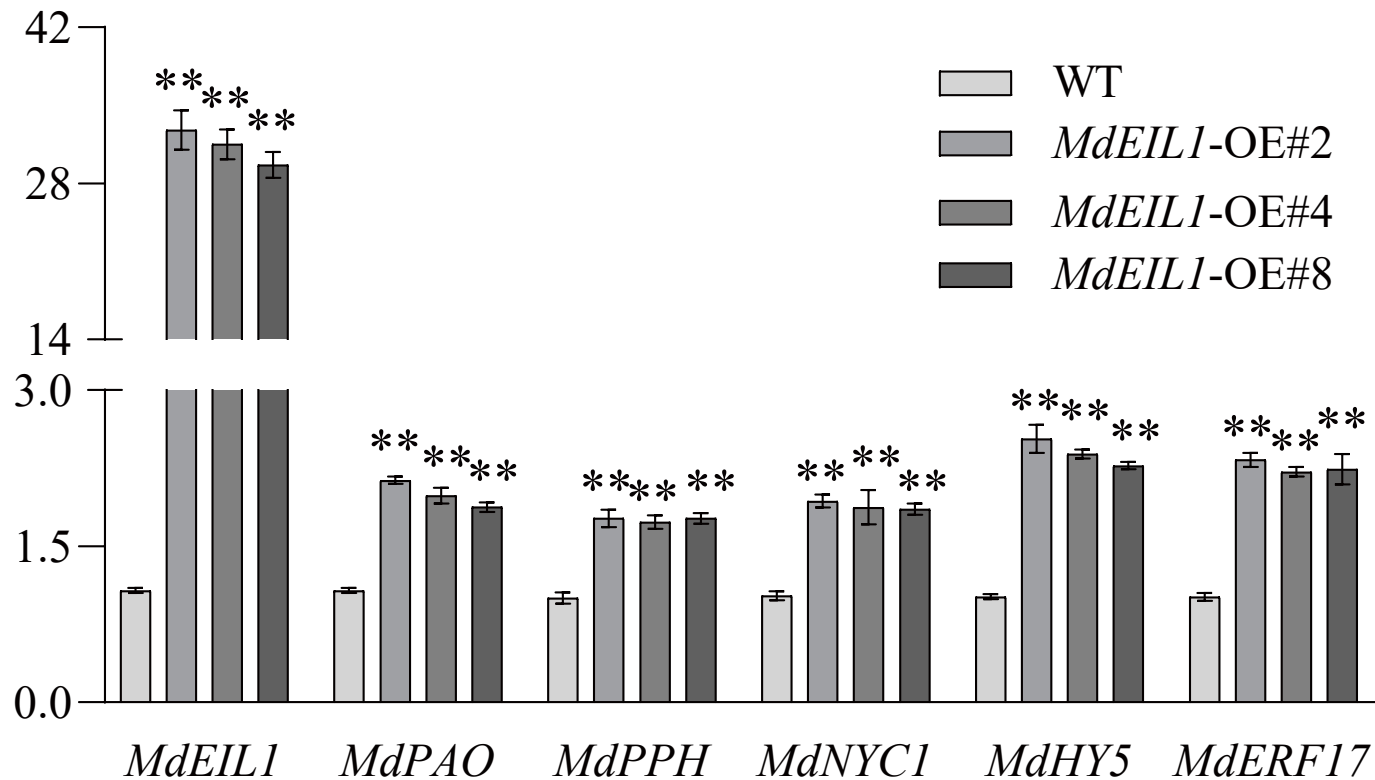

Supplement: Web_Material_uhae324 [file web_material_uhae324.zip › FigS2.pdf]

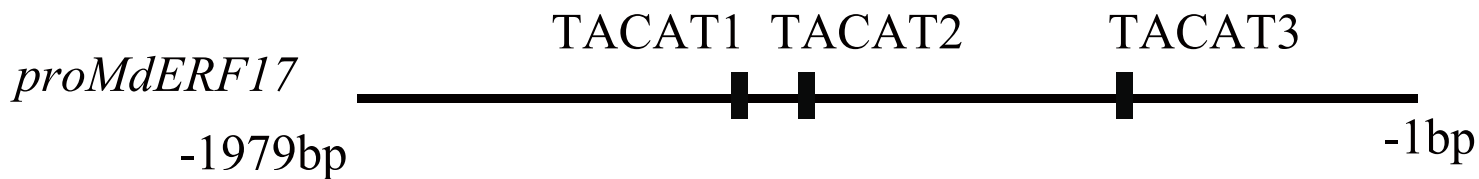

|            | TACAT1 | TACAT2 | TACAT3 |
|------------|--------|--------|--------|
| MdEIL1-HIS | +      | +      | +      |
| Hot probe  | +      | +      | +      |

Bound probe

Free probe

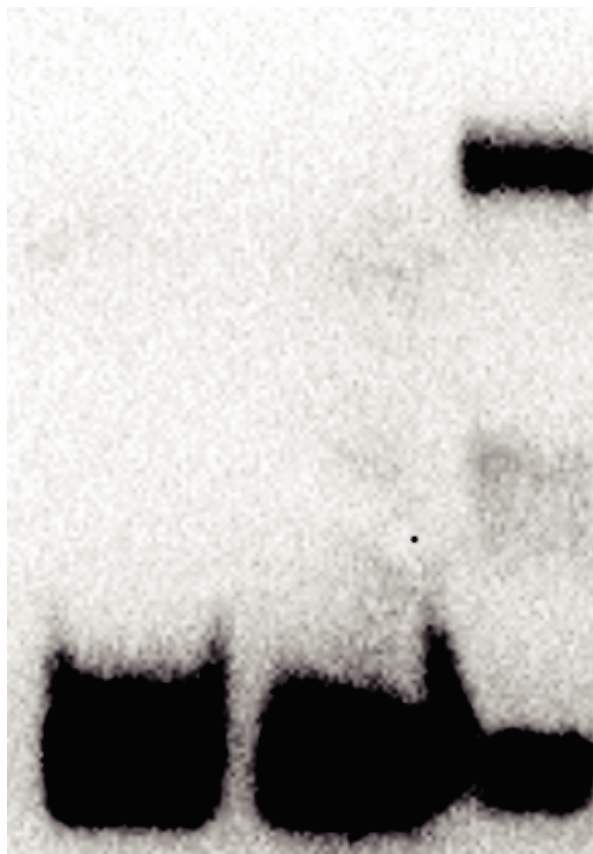

Supplement: Web_Material_uhae324 [file web_material_uhae324.zip › FigS3.pdf]

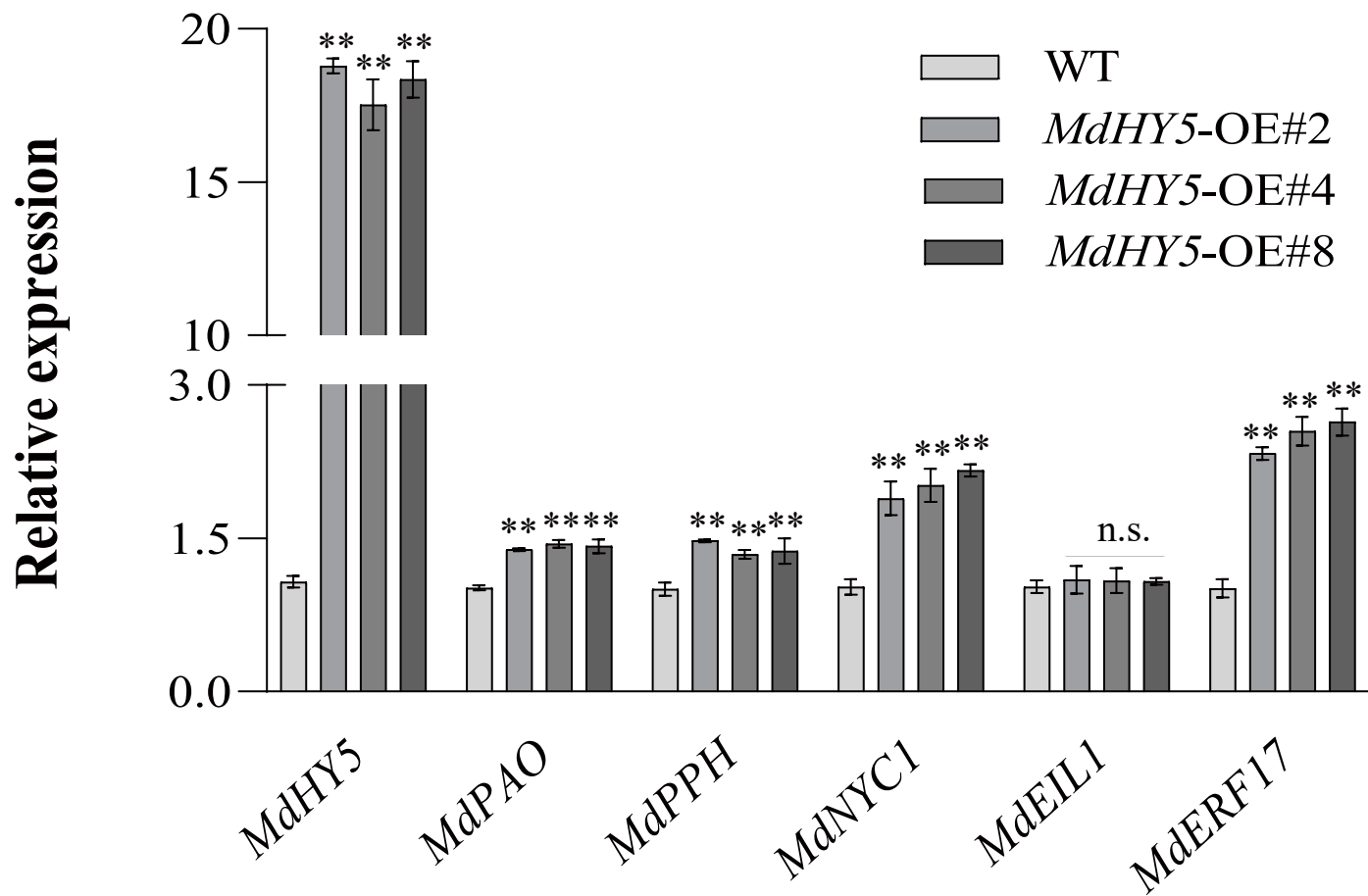

Supplement: Web_Material_uhae324 [file web_material_uhae324.zip › FigS4.pdf]

**a**

TRV+Eth

*MdHY5*-TRV+Eth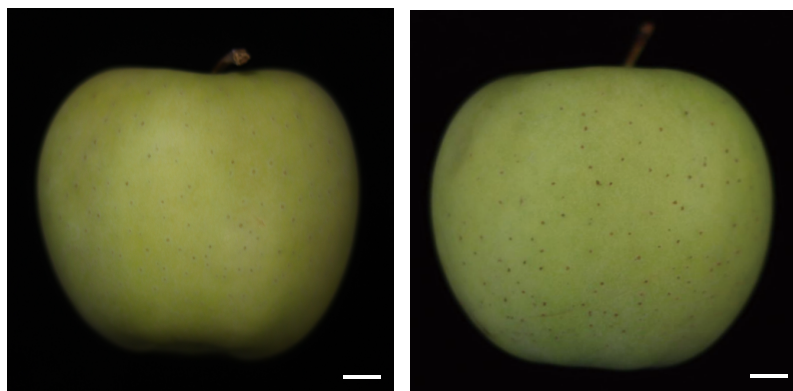**b**Chlorophyll contents  
mg/g FW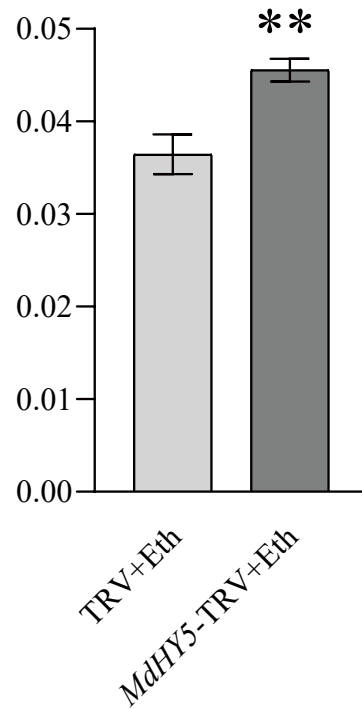**c**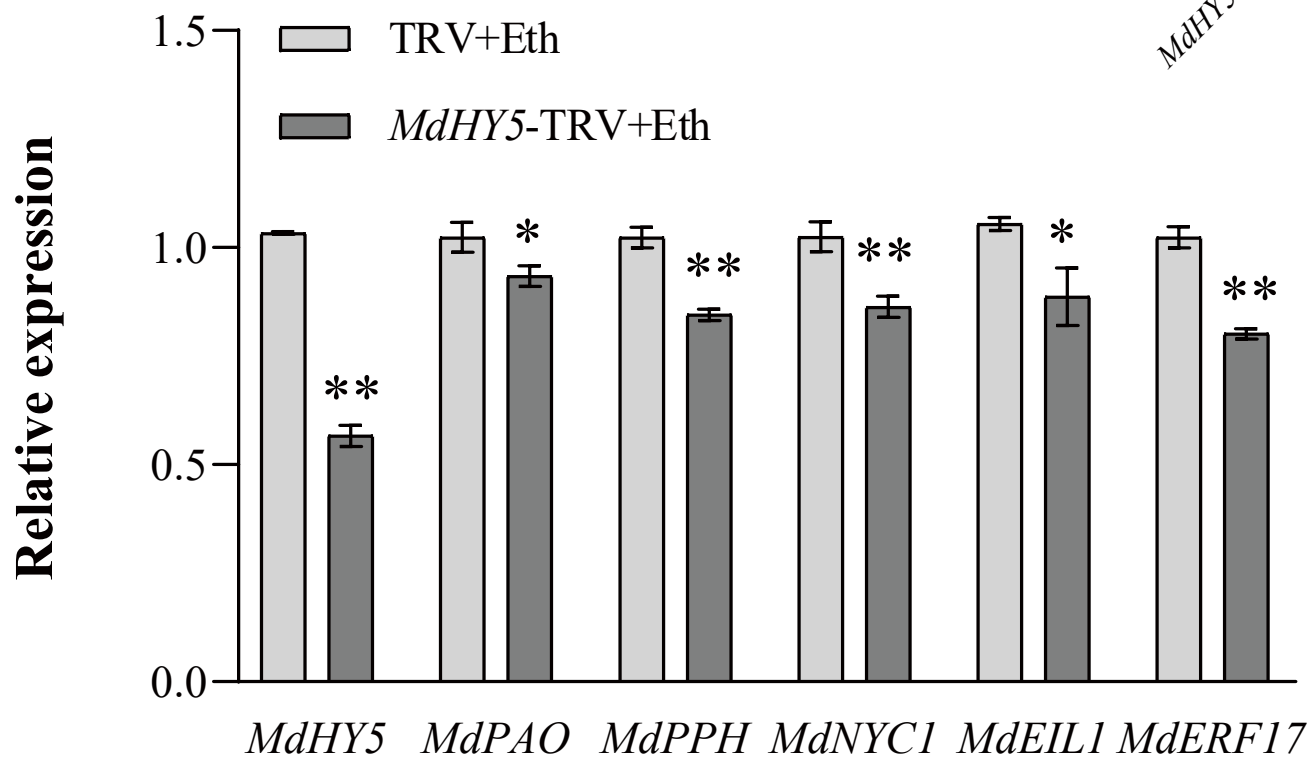

Supplement: Web_Material_uhae324 [file web_material_uhae324.zip › FigS5.pdf]

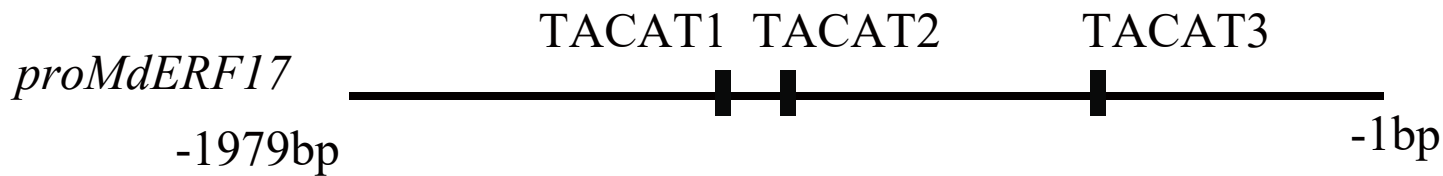

|            | TACAT1 | TACAT2 | TACAT3 |
|------------|--------|--------|--------|
| MdEIL1-HIS | +      | +      | +      |
| Hot probe  | +      | +      | +      |

Bound probe

Free probe

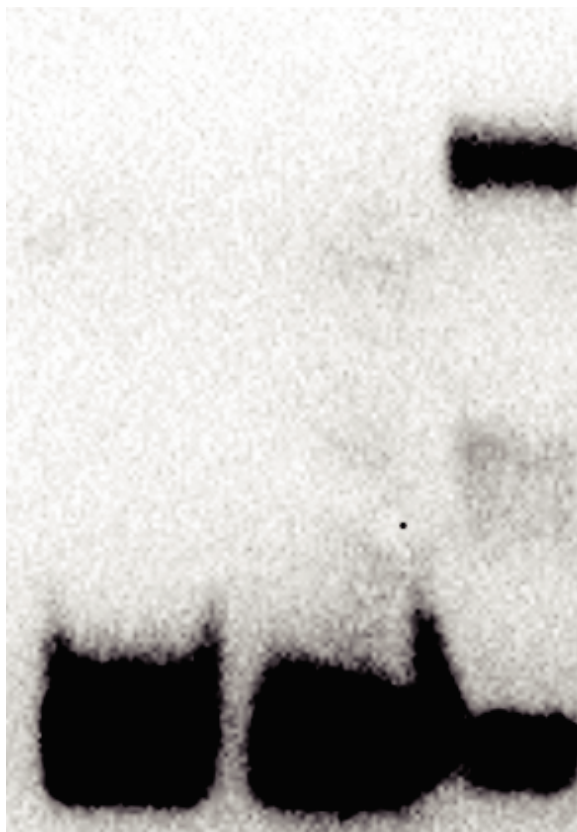

Supplement: Web_Material_uhae324 [file web_material_uhae324.zip › FigS6.pdf]

**a**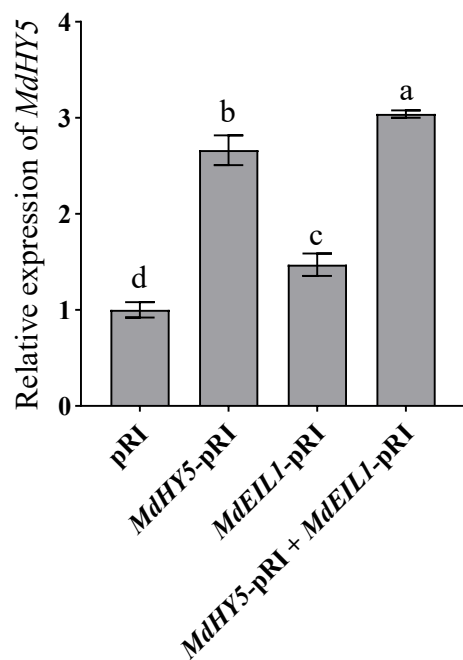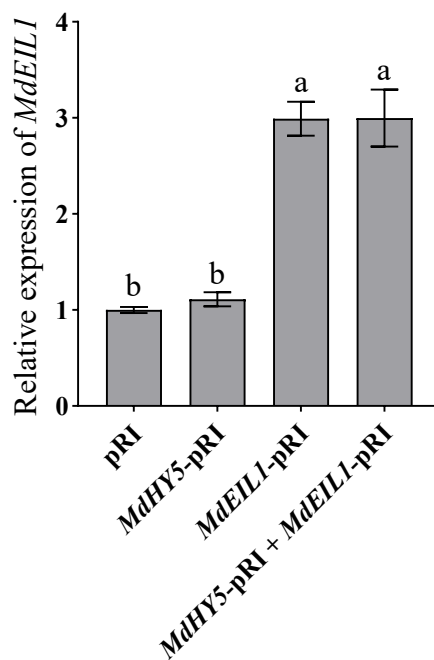**c**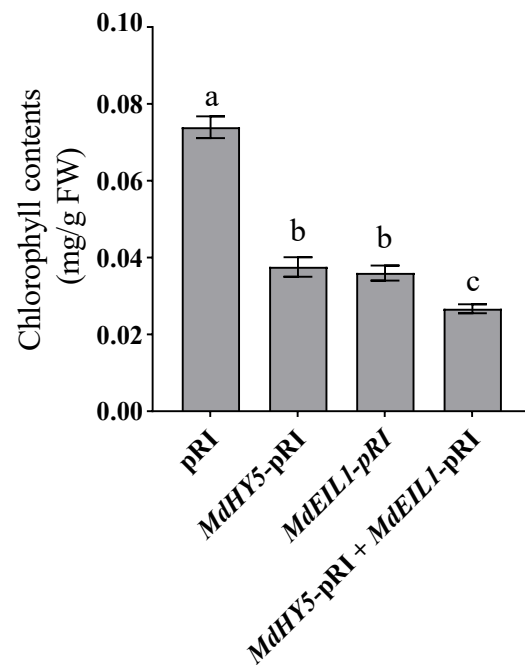**b**

pRI

*MdHY5*-pRI*MdEIL1*-pRI*MdEIL1*-pRI +  
*MdHY5*-pRI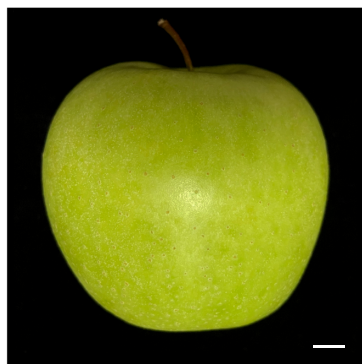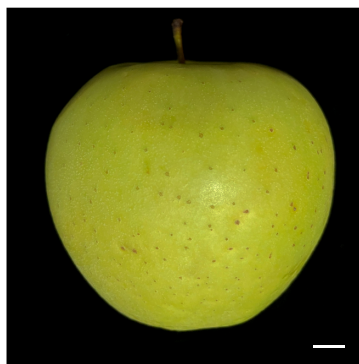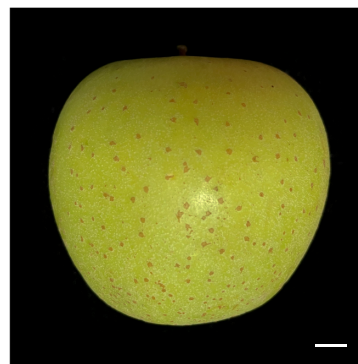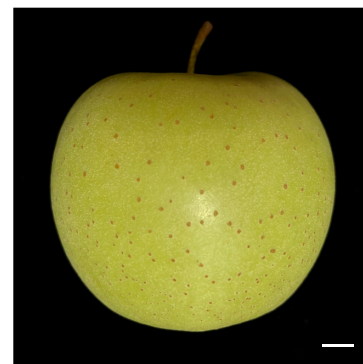

Supplement: Web_Material_uhae324 [file web_material_uhae324.zip › FigS8.pdf]
